# 基于HRCT亚厘米肺磨玻璃结节良恶性预测模型建立与验证

**DOI:** 10.3779/j.issn.1009-3419.2023.101.15

**Published:** 2023-05-20

**Authors:** CHEN Zhengwei, WANG Gaoxiang, WU Hanran, WU Mingsheng, WU Xianning, XU Meiqing, XIE Mingran

**Affiliations:** ^1^241001 芜湖，皖南医学院; ^1^Wannan Medical College, Wuhu 241001, China; ^2^230001 合肥，中国科学技术大学附属第一医院胸外科; ^2^Department of Thoracic Surgery, The First Affiliated Hospital of University of Science and Technology of China, Hefei 230001, China

**Keywords:** 高分辨率计算机断层扫描, 亚厘米肺磨玻璃结节, 良恶性病变, 预测模型, 列线图, High resolution computed tomography, Subcentimeter pulmonary ground glass nodules, Benign and malignant lesions, Prediction model, Nomogram

## Abstract

**背景与目的** 亚厘米磨玻璃结节（subcentimeter ground glass nodules, SGGNs）术前精准定性是临床工作的难点，目前关于SGGNs良恶性预测模型临床研究较少。本研究旨在基于高分辨率计算机断层扫描（high resolution computed tomography, HRCT）影像学特征与患者一般临床资料，帮助鉴别SGGNs良恶性病变，并构建风险预测模型。**方法** 回顾性分析2020年8月-2021年12月于中国科学技术大学附属第一医院接受手术切除并经组织学证实的483例SGGNs患者的临床资料，按7:3随机分配原则分为训练集（n=338）和验证集（n=145），根据术后组织学病理分为腺癌组和良性病变组。采用单因素分析和多因素Logistic回归分析独立危险因素和模型构建，受试者工作特征（receiver operator characteristic, ROC）曲线评估模型区分度，校准曲线评估模型的一致性，绘制临床决策曲线分析（decision curve analysis, DCA）评估模型的临床应用价值，并将验证集数据代入进行外部验证。**结果** 多因素Logistic分析筛选出患者的年龄、血管征、分叶征、结节体积和mean-CT值是SGGNs的独立危险因素，基于多因素分析结果构建列线图预测模型，ROC曲线下面积为0.836（95%CI: 0.794-0.879），最大约登指数所对应的临界值为0.483，此时敏感度为76.6%，特异度为80.1%，阳性预测值为86.5%，阴性预测值为68.7%。Bootstrap法抽样1,000次，校准曲线图预测的SGGNs良恶性风险与实际发生风险高度一致。DCA显示当预测模型概率的预概率为0.2-0.9，患者表现为正的净收益。**结论** 通过术前病史及术前HRCT检查指标确立SGGNs良恶性风险预测模型具有较好的预测效能与临床应用价值，列线图的可视化展现形式有助于筛选出SGGNs的高危人群，为临床决策提供支持。

## Abstract (en)

**Background and objective** Pre-operative accuracy of subcentimeter ground glass nodules (SGGNs) is a difficult problem in clinical practice, but there are few clinical studies on the benign and malignant prediction model of SGGNs. The aim of this study was to help identify benign and malignant lesions of SGGNs based on the imaging features of high resolution computed tomography (HRCT) and the general clinical data of patients, and to build a risk prediction model. **Methods** This study retrospectively analyzed the clinical data of 483 patients with SGGNs who underwent surgical resection and were confirmed by histology from the First Affiliated Hospital of University of Science and Technology of China from August 2020 to December 2021. The patients were divided into the training set (n=338) and the validation set (n=145) according to 7:3 random assignment. According to the postoperative histology, they were divided into adenocarcinoma group and benign lesion group. The independent risk factors and models were analyzed by univariate analysis and multivariate Logistic regression. The receiver operator characteristic (ROC) curve was constructed to evaluate the model differentiation, and the calibration curve was used to evaluate the model consistency. The clinical application value of the decision curve analysis (DCA) evaluation model was drawn, and the validation set data was substituted for external verification. **Results** Multivariate Logistic analysis screened out patients' age, vascular sign, lobular sign, nodule volume and mean-CT value as independent risk factors for SGGNs. Based on the results of multivariate analysis, Nomogram prediction model was constructed, and the area under ROC curve was 0.836 (95%CI: 0.794-0.879). The critical value corresponding to the maximum approximate entry index was 0.483. The sensitivity was 76.6%, and the specificity was 80.1%. The positive predictive value was 86.5%, and the negative predictive value was 68.7%. The benign and malignant risk of SGGNs predicted by the calibration curve was highly consistent with the actual occurrence risk after sampling 1,000 times using Bootstrap method. DCA showed that patients showed a positive net benefit when the predictive probability of the predicted model probability was 0.2 to 0.9. **Conclusion** Based on preoperative medical history and preoperative HRCT examination indicators, the benign and malignant risk prediction model of SGGNs was established to have good predictive efficacy and clinical application value. The visualization of Nomogram can help to screen out high-risk groups of SGGNs, providing support for clinical decision-making.

肺癌在全世界范围内死亡率居所有恶性肿瘤之首，在国内肺癌发病率和及死亡率依然在各类恶性肿瘤中位列第一^[1⇓-3]^，其中，腺癌是最常见的组织学类型。近年来，随着高分辨率计算机断层扫描（high resolution computed tomography, HRCT）广泛应用及定期体检的普及，亚厘米磨玻璃结节（subcentimeter ground glass nodules, SGGNs）的检出率不断攀升。其中部分SGGNs直径小、特征不典型，误诊为肺癌的假阴性率较高，易造成误诊、漏诊^[4,5]^。随着JCOG0802和CALGB140503等研究^[6,7]^生存数据的公布，对于<2 cm的早期肺癌或癌前病变，亚肺叶切除为优选策略，而SGGNs手术契机选择存在争议，因此在不延误肺癌诊断的前提下减少良性切除已成为研究热点。美国医师学会认为SGGNs外科干预前，建议先进行一段时间CT随访^[8]^，这一建议增加了医疗成本，增加了大量的辐射暴露，并给患者带来心理压力^[9,10]^。本研究旨在通过对SGGNs患者一般临床资料及HRCT影像学特征构建SGGNs良恶性预测模型，以期为临床决策提供支持。

## 1 资料与方法

### 1.1 研究对象

本研究回顾性分析2020年8月-2021年12月于中国科学技术大学附属第一医院接受手术切除且经组织学证实的483例亚厘米肺腺癌（n=255）和肺良性病变（n=228）的SGGNs患者的资料，其中包含纯GGNs 76例、混合GGNs 262例，按7:3随机分配原则分为训练集（n=338）和验证集（n=145）。纳入标准：（1）于我院行HRCT检查且结节平均直径≤1 cm；（2）符合2017年 Fleishner学会制定的肺结节处理指南^[8]^并接受手术治疗；（3）有术后病理证实；（4）术前胸部CT扫描与手术间隔<2周。排除标准：（1）影像学结节平均直径>1 cm；（2）病理诊断为微浸润性腺癌（microinvasive adenocarcinoma, MIA）或原位腺癌（adenocarcinoma in situ, AIS）；（3）影像学图像不清晰，有明显伪影；（4）病历资料不完善。本研究经中国科学技术大学附属第一医院伦理审查委员会批准（No.2023-RE-097），手术患者均签署知情同意书。

### 1.2 研究方法

受试者均于我院行HRCT检查，将患者影像学检查图像传输至配套工作站（安徽科大讯飞肺部医学影像辅助诊断软件）以计算结节体积max-CT值、mean-CT值、min-CT值、结节体积及结节最大直径。通过在HRCT平面上选取实性成分面积最大、密度最高处任意3个感兴趣区（region of interest, ROI），并测量出其实性成分最大径。本文实性成分占比（consolid/tumor ratio, CTR）由结节实性成分最大径和结节最大径比值得出。由2名3年以上工作经验医师对软件筛选结果单独进行判断，若结果出现争议则由2名医师进行综合判读。其中主要观察受试者结节部位、结节大小、结节边界是否清晰、血管征、胸膜牵拉征、空泡征、毛刺征、结节平均大小、结节平均体积、结节实性成分最大径、结节最大径、CTR；同时临床上收集患者临床病历信息。

手术标本的组织病理学检查由2名接受过胸部病理诊断亚专科培训的病理医生（1名工作经验5年以上，1名工作经验10年）进行，对病理切片不满意或争议时，结合术前影像学报告，讨论后综合评估诊断。切除肺病变组织按照2011年国际肺癌研究协会/美国胸科学会/欧洲呼吸学会分类体系和2015年世界卫生组织肺肿瘤分类进行分类^[11]^。

### 1.3 统计学方法

应用SPSS 26.0进行数据分析，P<0.05为差异有统计学意义。应用卡方检验或秩和检验比较两组患者临床病历资料，应用单因素分析评估SGGNs与肺腺癌病变的关联性，仅对训练集中单因素分析有统计学意义的因素进一步纳入多因素二元Logistic回归分析，以寻找独立危险因素（包括临床因素、主观HRCT特征、mean-CT值）。将最终独立危险因素引入R软件（The R Foundation for Statistical Computing, Vienna, Austria）4.2.2版，采用“pROC”进行受试者工作特征（receiver operator characteristic, ROC）分析和DeLong检验。Nomogram用“rms”完成，决策曲线分析（decision curve analysis, DCA）用“rmda”完成。曲线下面积均采用自举偏差校正的95%CI。所有的统计检验为双侧检验。同时建立ROC在训练集和验证集中的表现，曲线下面积以评估此模型；为了评估Nomogram的临床应用价值，使用临床校准曲线展示预测数据和实际数据之间拟合情况；通过计算一系列阈值概率的净收益，使用所有数据集进行DCA。

## 2 结果

### 2.1 两组患者临床及影像学资料分析

本研究训练集中共338例患者，其中男性163例，女性175例，良性组和腺癌组患者的性别、吸烟史、是否位于上叶、结节性质、空泡征、胸膜牵拉、CTR、实性成分最大径、结节最小CT值差异无统计学意义（P>0.05），患者年龄、家族肿瘤史、边界是否清晰、血管征、分叶征、毛刺征、结节平均直径大小、结节体积、实性成分最大径、结节max-CT值、mean-CT值差异有统计学意义（P<0.05），见表1。

**表1:** 患者临床特征的单因素分析（训练集）

Variable	Benign (n=171)	Adenocarcinoma (n=167)	χ^2^/Z	P
Gender			χ^2^=2.692	0.101
Male	90	73		
Famale	81	94		
Age (yr)			χ^2^=7.789	0.005
<60	134	108		
≥60	37	59		
Smoking			χ^2^=0.294	0.587
Yes	25	28		
No	146	139		
Tumor history			χ^2^=8.39	0.004
Yes	0	8		
No	171	159		
Upper lobe			χ^2^=3.018	0.082
Yes	76	90		
No	95	77		
Density			χ^2^=0.143	0.706
pGGNs	37	39		
mGGNs	134	128		
Boundary clear			χ^2^=9.939	0.002
Yes	70	97		
No	101	70		
Vascular sign			χ^2^=50.146	<0.001
Yes	23	82		
No	148	85		
Pleural sign			χ^2^=2.194	0.139
Yes	40	61		
No	131	106		
Vocule sign			χ^2^=3.41	0.065
Yes	17	28		
No	154	139		
Lobulation sign			χ^2^=59.585	<0.0001
Yes	25	91		
No	146	76		
Spicule sign			χ^2^=5.314	0.021
Yes	31	48		
No	140	119		
CTR			χ^2^=0.712	0.399
0-0.5	64	70		
0.5-1	107	97		
Mean size^a^	9 (7, 10)	10 (8, 10)	Z=-4.759	<0.001
Nodule volume^a^	389 (257, 560)	534 (418, 624)	Z=-5.987	<0.001
Solid max^a^	5.7 (2.0, 7.9)	6 (1.1, 8.9)	Z=-0.69	0.49
Max-diameter^a^	9 (7, 10)	10 (8, 11)	Z=-4.754	<0.001
Mean-CT (HU)^a^	-326 (-575.4, -326.0)	-420 (-579, -288)	Z=-2.341	0.019
Max-CT (HU)^a^	-120 (-350, 30)	-283 (-409, -128)	Z=-4.824	<0.001
Min-CT (HU)^a^	-580 (-749, -340)	-568 (-651, -419)	Z=-2.341	0.140

^a^Data is represented by median (P_25_, P_75_); pGGNs: pure ground glass nodules; mGGNs: mixed GGNs; CTR: consolid/tumor ratio; mean-CT: mean computed tomography.

### 2.2 病理诊断结果

根据术后病理诊断分为肺腺癌组和肺良性病变组；训练集中肺腺癌组共167例，包括浸润性肺癌（145例）、黏液腺癌（10例）、低分化腺癌（12例）等；肺良性病变组共171例，包括非典型腺瘤样增生（14例）、肺肉芽肿（19例）、错构瘤（21例）和肺炎性病变（117例）。

### 2.3 多因素分析

将单因素分析有意义的结果纳入多因素分析显示：患者年龄（P=0.049）、血管征（P<0.001）、分叶征（P<0.001）、结节体积（P=0.018）、肺结节max-CT值（P<0.001）、mean-CT值（P<0.001）是亚厘米肺腺癌与肺良性病变的独立危险因素（表2）。

**表2:** 患者临床特征的多因素分析（训练集）

Variable	P	Exp(B)	OR (95%CI)
Age	0.049	1.928	1.002-3.708
Mean size	0.843	1.059	0.603-1.858
Boundary clear	0.244	0.687	0.366-1.292
Vascular sign	<0.001	7.091	3.436-14.636
Lobulation sign	<0.001	9.769	4.742-20.127
Spicule sign	0.336	1.441	0.685-3.029
Max-diameter	0.988	1.003	0.628-1.604
Nodule volume	0.018	0.996	0.992-0.999
Max-CT (HU)	<0.001	1.015	1.009-1.020
Mean-CT (HU)	<0.001	0.989	0.984-0.994

Considering the collinearity of max-CT value and mean-CT value, mean-CT was finally included in the model construction.

### 2.4 列线图模型构建

根据表2结果构建SGGNs良恶性预测模型列线图，由于结节max-CT值与mean-CT值存在共线性。因此，最终将mean-CT值等5个独立危险因素纳入列线图的构建（图1）。根据列线图模型中各个风险因素对结局变量的影响程度，对每个变量不同水平进行打分（即当年龄≥60岁得20分，血管征得42.5分，分叶征得45分，结节体积和mean-CT值数值对应列线图相应的分数），然后将各个变量得分相加得到总分；最后通过总评分计算结局事件发生概率，从而计算该结果的预测值大小。

**图1:**
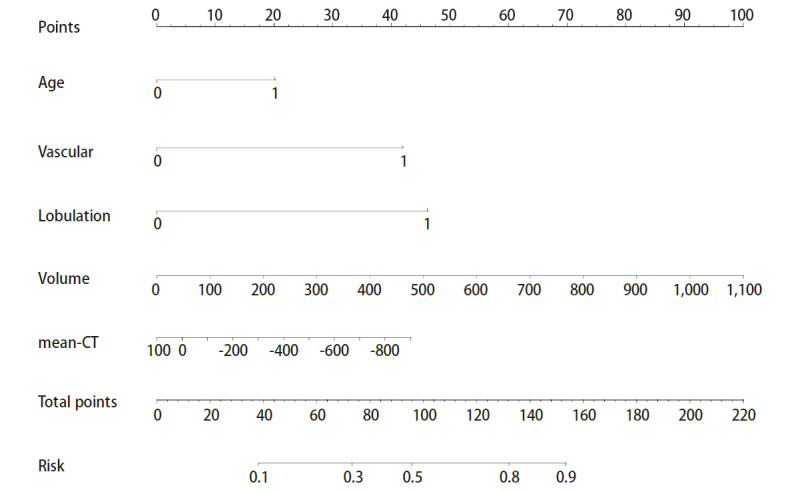
SGGNs良恶性预测模型的列线图分析

### 2.5 预测模型验证与效能评价

预测模型的区分度：将多因素分析结果带入该模型，预测良恶性的ROC曲线下面积为0.836（95%CI: 0.794-0.879），最大约登指数所对应的临界值为0.483，此时敏感度为76.6%，特异度为80.1%，阳性预测值为86.5%，阴性预测值为68.7%。利用外部验证集数据验证该模型，得到ROC曲线下面积为0.728（95%CI: 0.646-0.810）（图2），提示本预测模型在训练集和验证集都具有较好的区分度。

**图2:**
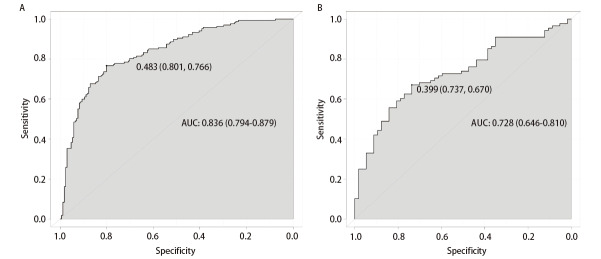
SGGNs良恶性预测模型的ROC分析。A：训练集；B：验证集。

预测模型的校准度：训练集和验证集通过加强Bootstrap法重复抽样1,000次进行内外部验证，校准曲线显示的预测值与实际值高度吻合（图3），平均绝对误差为训练集（P=0.008）、验证集（P=0.019），提示使用校准曲线发现训练集和验证集中的预测数据和实际数据之间有显著的联系，校准曲线图预测的SGGNs良恶性预测风险与实际发生风险高度一致。

**图3:**
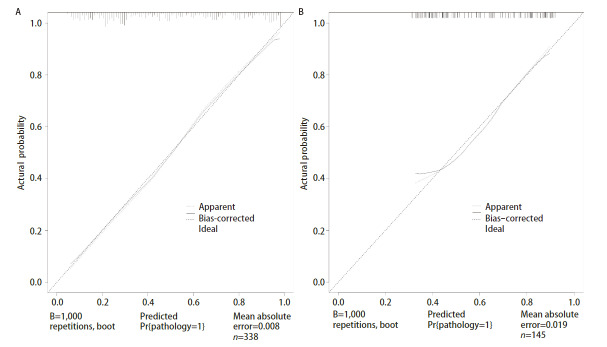
SGGNs良恶性预测模型的校准曲线分析。A：训练集；B：验证集。

预测模型的适用度：DCA显示模型的阈概率为0.2-0.9（图4），模型表现为正的净收益，图中两条曲线代表两种极端情况，标“无”的横线表示所有患者均为SGGNs，且不进行干预，净收益为0，标“全部”的斜线表示所有患者均SGGNs，并实施干预所获得的净收益。红色的曲线是采用列线图预测模型下患者所获得的临床净收益。

**图4:**
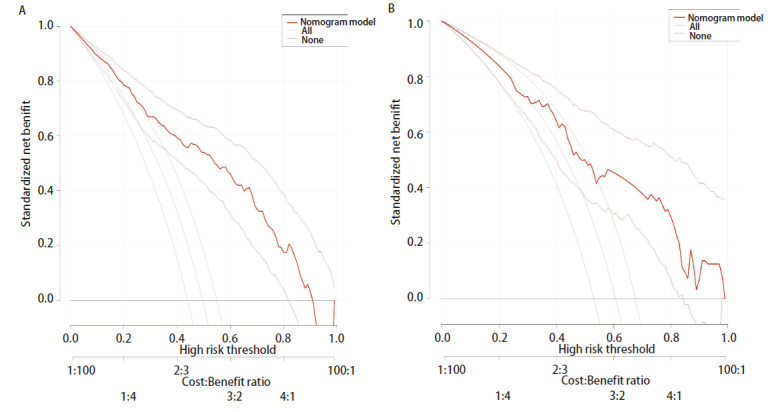
SGGNs良恶性预测模型的临床DCA。A：训练集；B：验证集。

## 3 讨论

SGGNs病理可以是腺癌、原位癌、不典型腺瘤样增生或良性病变，如肺纤维化、炎性假瘤。目前，腺癌是非小细胞肺癌中最常见的原发性肺恶性肿瘤，一经明确，应积极治疗^[12]^。研究^[13]^发现，30%-90%的纯GGNs在随访3个月后消失，这些消失的pGGNs考虑是炎症。虽然外科切除可以识别和治疗更多的早期肺癌，但它也会使部分患有良性病变的患者面临侵入性手术风险。此外，随访观察可能会延误恶性肿瘤的治疗进展^[14]^，这对于SGGNs的定性诊断造成很大困扰。本研究旨在开发一种基于HRCT影像学特征新的诊断模型Nomogram，在临床上为SGGNs良恶性鉴别问题提供便利，为临床决策提供支持。

分叶征和血管征是肺癌常见和重要影像学特征。Lv和Qi等^[15,16]^分别通过回顾性分析浸润性肺腺癌和表现为GGNs的浸润前病变之间的CT扫描特征，结果显示分叶征和血管征是显著的区分因素，这与我们的研究结果一致，我们分析可能与以下原因有关：（1）分叶征在良性病变中相对少见，而肿瘤细胞往往呈多核发病及相互融合或病灶内肿瘤细胞生长速度过快，肿瘤生长被邻近的肺间质所阻滞，从而导致结节内纤维组织收缩，故分叶征常预示着恶性肿瘤^[17]^。（2）血管集束征常与病灶中的纤维成分牵拉、血管及肿瘤细胞释放大量血管内皮生长因子有关，生成的大量新生血管为肿瘤的生长、浸润及转移提供了重要的营养物质及路径^[18]^。虽然，很多炎性结节也会有血管穿行，“血管穿行”并不是恶性结节独立危险因素，但当单支或多支血管穿过或向GGNs病灶集中聚拢，并呈现出扭曲、僵直形态时，提示该GGNs为恶性肿瘤的可能性大。

目前，各种CT参数在GGNs诊断中的应用逐渐被重视，特别是通过对CTR值、mean-CT值、max-CT值、pGGNs及mGGNs中实性成分大小的研究^[19]^发现，Zhang等^[20]^通过回顾性分析上海交通大学医学院上海总医院接受肺切除手术并有病理证实的314例患者共358个GGNs的资料，发现GGNs的mean-CT值和密度偏差有助于识别浸润性病变。本研究也证实了结节max-CT值、mean-CT值为GGNs良恶性的独立危险因素，我们分析原因可能是SGGNs在影像学上可以发现的异常是“磨玻璃”区域，随着时间的推移，其大小会增加，形成更坚固的成分，然后变成侵袭性肿瘤，而CT值越高，提示该结节密度越高，坚固的成分越高，提示其侵袭性越高^[21]^。目前有研究^[22,23]^表明CTR>0.5常提示恶性程度增加，本研究将亚厘米肺结节CTR和实性成分最大径也纳入研究中，结果提示CTR与实性成分最大径在良恶性鉴别方面无统计学差异。有学者^[24]^认为CTR、实性成分最大径与CT值存在相同病理基础，所以理论上实性成分越大、CTR值越大、mean-CT值越高，结节恶性程度越高。笔者考虑造成差异主要原因是：（1）两者测量方法存在明显差异，CTR利用ROI并手动测量，而mean-CT值通过医学影像辅助诊断软件自动计算，使结果更加精确；（2）本研究对照组为肺良性病变，其中包括错构瘤、肺肉芽肿，其CTR接近1，而病理结果却提示良性，导致结果存在统计学差异。Wu等^[25]^通过回顾性分析中国科学技术大学附属第一医院胸外科接受肺切除手术并病理证实为肺GGNs的129例患者的资料，通过比较max-CT值、mean-CT值、实性成分最大径、肿瘤最大径、CTR，发现结节的mean-CT值对预测GGNs的恶性程度的准确性相对最高，是鉴别良恶性的优秀指标。

另外，毛刺征在各种预测模型中均为独立危险因素，而在本研究中，毛刺征不是独立危险因素，笔者考虑造成差异的主要原因是：（1）本研究对照组为肺良性病变，包括非典型腺瘤样增生、肺肉芽肿、错构瘤、炎性假瘤的患者较多，这些SGGNs在影像学上也可出现毛刺征^[26]^，因此这些差异是可接受的；（2）本研究仅纳入经组织学证实为亚厘米肺腺癌或肺良性病变的手术切除SGGNs，我们的结节样本可能倾向于形态上更明显或更具侵略性的恶性结节。相反，这种纳入标准保证了结节的病理均匀性。

关于肺结节风险预测模型较多，其制作的方式均为类似的，但仍有部分文献在对模型进行构建并未进行验证或验证分析发现曲线下面积并不完善，原因可能是研究设计及方法存在缺陷。Xu等^[27]^通过回顾性分析2015年3月-2018年3月在北京友谊医院接受术前胸部CT检查的单发肺结节患者（n=373）的非增强薄层CT特征，分别开发出T1a、T1b和T1c良恶性预测模型，结果显示T1a预测模型的曲线下面积、准确性、敏感性和特异性明显优于T1b和T1c良恶性预测模型，然而设计模型并未进行验证。本研究通过对训练集进行多因素回归分析，采用ROC曲线、DCA曲线、校准曲线分别对预测模型进行评价，最终将患者的年龄、血管征、分叶征、肿瘤体积、mean-CT值纳入列线图模型的构建，其性能在训练集中曲线下面积为0.836（95%CI: 0.794-0.879），此时，最大约登指数所对应的临界值为0.483，此时敏感度为76.6%，特异度为80.1%，阳性预测值为86.5%，阴性预测值为68.7%。将验证集中145例患者的数据代入模型绘制ROC曲线，其曲线下面积为0.728，表明本模型具有较好预测价值，通过加强Bootstrap法重复抽样1,000次进行内外部验证，校准曲线高度拟合显示预测数据与实际数据发生风险高度一致。

综上所述，基于HRCT影像学特征及一般临床资料制作的SGGNs列线图预测模型提高了肺结节良恶性鉴别的正确率，是减少SGGNs过度治疗的有效工具。由于本研究是单中心回顾性研究，这些结果可能会受到选择偏倚的影响，进一步的研究应该从多中心入组更多的患者，以便更好地验证SGGNs良恶性预测模型。
